# Real-world disparities and ethical considerations with access to CFTR modulator drugs: Mind the gap!

**DOI:** 10.3389/fphar.2023.1163391

**Published:** 2023-03-27

**Authors:** M. Zampoli, B. M Morrow, G Paul

**Affiliations:** ^1^ Department of Paediatrics and Child Health and Red Cross War Memorial Children’s Hospital, University of Cape Town, Cape Town, South Africa; ^2^ Division of Pulmonary Medicine, Nationwide Children’s Hospital, Columbus, OH, United States

**Keywords:** cystic fibrosis, low-middle income countries, disparity, CFTR modulator drugs, ethics

## Abstract

The third Sustainable Development Goal (SDG), to ensure healthy lives and promote well-being for all at all ages, has particular relevance and implementation challenges amongst people living with rare diseases such as cystic fibrosis (CF). Although the treatment and projected outcome of CF has significantly improved with the advent of CF transmembrane conductance regulator protein modulator (CFTRm) therapy, there remains significant global inequality with regards to access to these life-saving and life-altering drugs. Elexacaftor, tezacaftor, and ivacaftor (ETI) triple combination therapy, first licensed in the United States in 2019, has rapidly become the standard of care for children aged 6 years and older in most high-income countries for individuals with CFTR variants responsive to ETI. Negotiated agreements for access to ETI are currently in place in North America,Europe, Israel ,Australia and New Zealand. However, less priority has been given to negotiate agreements for access to CFTRm in low-middle income countries(LMIC) with significant CF populations such as Central and South America, India, the Middle East, and Southern Africa. These countries and individuals living with CF are therefore effectively being left behind, in direct conflict with the stated principle of the 2030 SDGs. In this review, we highlight the current global inequity in access to CFTRm drugs and its impact on widening disparities between high-income countries and LMIC in CF outcomes and survival. We further discuss the reasons for this inequity and explore the ethical- and human rights-based principles and dilemmas that clinicians, families, governments, and healthcare funders must consider when prioritizing fair and affordable access to expensive CFTRm drugs. Lastly, we propose possible solutions to overcoming the barriers to accessing affordable CFTRm drugs in LMIC and illustrate with examples how access to drug therapies for other conditions have been successfully negotiated in LMIC through innovative partnerships between governments and pharmaceutical industries.

## Introduction

The advent of cystic fibrosis transmembrane conductance regulator modulator (CFTRm) drugs has revolutionized the treatment of cystic fibrosis (CF) in individuals with drug-responsive CFTR variants. Elexacaftor, tezacaftor, and ivacaftor (ETI) triple combination therapy, first licensed in the United States (US) in 2019, has rapidly become standard of care in most high-income countries (HIC) for people with CF (pwCF) aged 6 years and older with CFTR variants responsive to ETI, the commonest being F508del. Beyond clinical trials, real-world data on outcomes in pwCF on ivacaftor, the first CFTRm licensed more than a decade ago, provide compelling evidence over time of improved outcomes in nutrition, pulmonary exacerbations, and lung function. Furthermore, risk for mortality and lung transplantation are also significantly reduced (Balfour-Lynn and King, 2022; Gifford et al., 2022; Regard et al., 2022). Although long-term, real-world data are lacking for ETI, similar outcomes are reported and projected for pwCF receiving ETI, including those with advanced lung disease (Benden and Schwarz, 2021; Balfour-Lynn and King, 2022; Gifford et al., 2022; Keogh et al., 2022; Regard et al., 2022; McCoy et al., 2023). The outlook and long-term prognosis, including survival, of pwCF has without doubt substantially improved for those eligible for CFTRm and have access to these drugs, especially if initiated at a younger age when end-organ disease is not established yet. Ivacaftor is currently licensed from age 6 months, and results of phase III clinical trials for ETI in children aged 2–5 years are expected to be released soon (NCT04537793).

Worldwide, 82% of people diagnosed with CF have at least one copy of F508del and are thus eligible for ETI (Lopes-Pacheco, 2020). However, the global demography of CFTR mutations is largely determined by race and ethnicity, with the proportion of F508del in at least one allele ranging between 80% and 90% in North America, Western Europe, and Australia, less than 20% in Turkey, and extremely rare in people with sub-Saharan African or Asian ancestry (Stewart and Pepper, 2017; Lopes-Pacheco, 2020; McGarry et al., 2022). In people with African ancestry, the class-I variant 3120+1G>A is the most commonly reported mutation, present in a homozygous state in 56% of black South Africans with CF (Zampoli et al., 2021). Race and ethnic diversity are thus important biological factors determining eligibility for CFTRm, strongly favoring populations where F508del is common.

The third Sustainable Development Goal (SDG), to ensure healthy lives and promote well-being for all at all ages, enshrined in the pledge to “leave no one behind,” has particular relevance and implementation challenges amongst people living with rare diseases such as CF (United Nations Committee for Development Policy, 2018). There are significant global disparities and inequalities in accessing expensive life-saving and life-altering CFTRm drugs, with only an estimated 12% of the world’s total CF population receiving this therapy (Guo et al., 2022a). This inequality is likely to accelerate a widening gap in CF care and outcomes between high income countries (HIC) and low-middle income countries (LMIC).

## Worldwide disparities in CF care and access to CFTRm drugs

According to the WHO, social determinants of health (SDOH) can be more important than healthcare services or lifestyle choices. Worldwide, irrespective of income or infrastructure, health and illness follow a social gradient: *the lower the socioeconomic position*, *the worse the health*. Non-medical factors and SDOH potentially impact 30%–55% of health outcomes (Hosseinpoor et al., 2012; World Health Organisation, 2023). In diseases such as CF, where exceptional standards of care have been established, race, ethnicity, and SDOH are important factors affecting CF outcomes in all facets of diagnosis, management, and prognosis. In many LMIC and among marginalized populations within developed nations, there is notable ascertainment bias due to lack of awareness of CF, prioritization on endemic diseases, lack of CF registries, absent or limited newborn screening (NBS) protocols, or NBS panels analyzing mutations that are not relevant to that population (Scotet et al., 2020; da Silva Filho et al., 2021). Limited access to sweat testing and full mutation analyses impedes diagnostic confirmation and hence knowledge of CFTRm eligibility.

**FIGURE 1:**
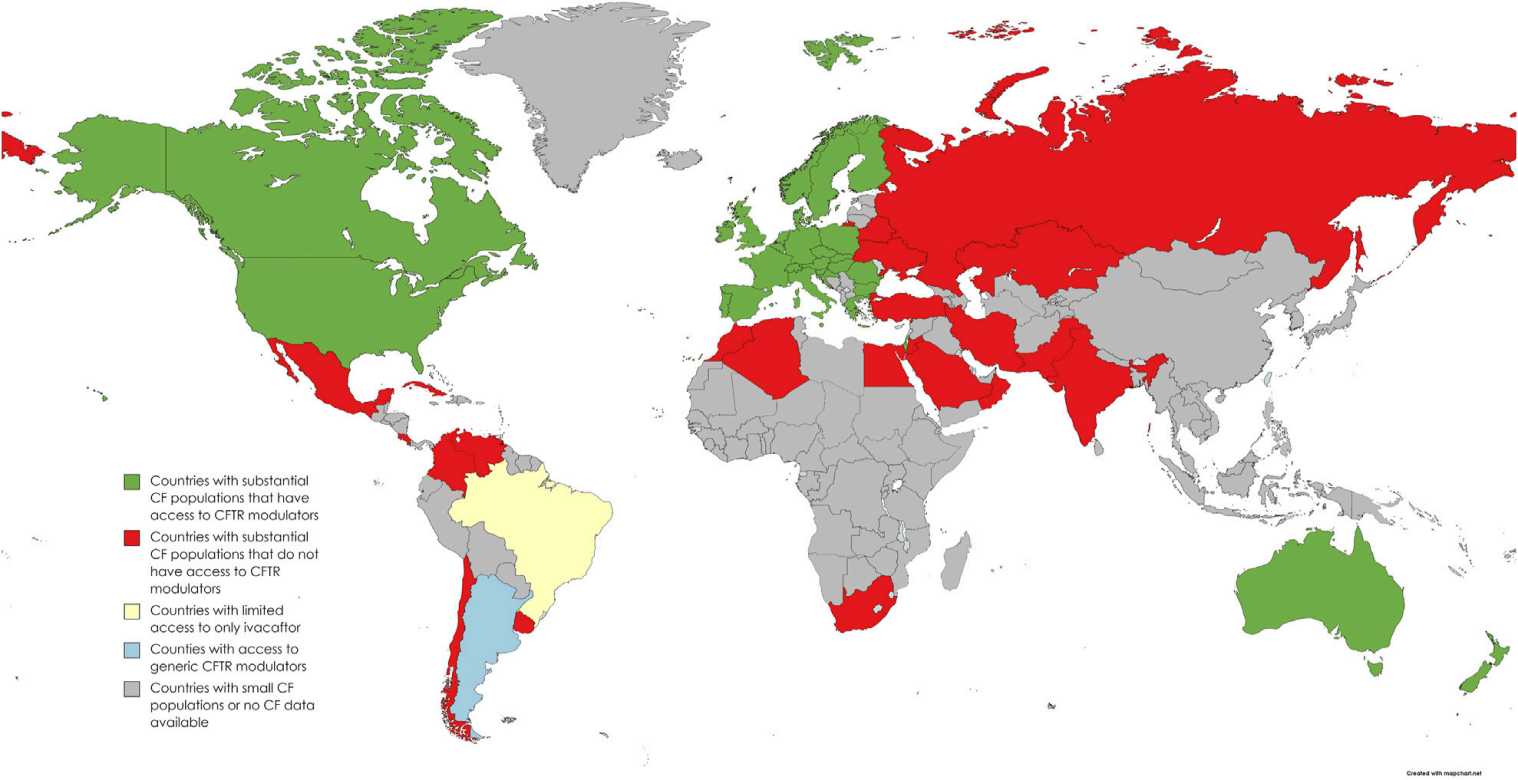
World map distribution of countries with access to CFTR modulator drugs or negotiated agreements in place (adapted from Guo et al. (2022a) and personal communications with “Vertex Save Us” campaign).

The estimated number of confirmed pwCF from 94 countries worldwide is 105,000, with no information available from the other 109 nations (Guo et al., 2022a). The estimated global disease burden is 144,606–186,620 based on surveys, registry data, and extrapolated data. Worldwide, 40 countries have at least one CFTRm approved, but the majority are HIC with predominantly White race populations in North America, Europe, Israel, Australia and New Zealand (Figure 1). According to the annual US CF registry report, 87.6% of 25,497 pwCF eligible for CFTRm drugs in the US were receiving CFTRm drugs in 2021 (CFF, 2022). This is in stark contrast to the global picture where only 19,516 (12%) of the estimated 162,428 world CF population, both diagnosed and undiagnosed, are receiving CFTRm drugs (Guo et al., 2022b).

Unequal availability of CFTRm will only widen existing disparities in disease burden and outcomes in LMIC which have weaker healthcare infrastructure compared to HIC. Even though the reported prevalence of CF is low in many LMIC, when extrapolated to actual population data, absolute numbers of pwCF may be very high. For example, in India with a population of 1.4 billion, actual numbers of pwCF could range between 35,000 and 140,000 based on reported prevalence of 1: 10,000–1:40,000. Similarly, in 2021, there were 23 million live births with an estimated CF incidence between 680 and 2,700 (Kapoor et al., 2006; Singh et al., 2020). Without more accurate information on CFTRm-eligible mutations, it is difficult to estimate how many patients could benefit from CFTRm worldwide.

## The impact of race and ethnicity on CF care and access to CFTRm

Race and ethnicity are fundamental components of a person’s identity. While race classifications are often based on physical and biogenetic characteristics, especially skin color, ethnic distinctions focus on cultural characteristics such as language, history, religion, and custom. There is no genetic basis for race, and evidence from the Human Genome Project and 1000 Genomes Project revealed only 0.1% difference among races with probable shared ancestry (Collins et al., 2003; Genomes Project et al., 2015). While the realization of racial and ethnic diversity should be embraced, race has historically been the focus of disparities ever since the beginning of civilization and significantly impacts SDOH, leading to prejudice, inequity, and discrimination.

According to the 2021 US CF registry data, about 18% are self-reported as “non-white” people (CFF, 2022). Even in a well-resourced nation with access to the best possible CF care, delayed diagnosis and delayed time to first assessment after a positive NBS were noted among infants from minority communities with worse pulmonary or nutritional morbidity. There is clear demonstration of lower lung function, higher mortality rates, and higher rates of acquiring pulmonary infections related to delayed diagnosis and limited access to care, limited health literacy, and ill effects of systemic racism. Furthermore, low-income groups experience differential exposure to tobacco smoke, access to transplantation, inclusion in clinical trials, mental health issues—all factors that impede adherence to recommended clinical care. SDOH directly and indirectly impact CF outcomes through policy, socioeconomic status, and race/ethnicity (Quittner et al., 2010; McGarry and McColley, 2021; Oates and Schechter, 2021; McGarry et al., 2022). This exciting era of CFTRm has unfortunately only exposed the existing disparities. The prevalence of F508del mutations among pwCF from minority communities in the US is lower with significant disparities in CFTRm eligibility: 92% in White people *versus* 70%–75% in African–Americans and Hispanic pwCF, respectively (McGarry and McColley, 2021). Similarly, emerging data from many LMIC reveal mutational heterogeneity, with lower F508del allele frequency (da Silva Filho et al., 2021; Singh et al., 2015; Vaidyanathan et al., 2022).

## Ethical- and human rights-based framework: The case for CFTRm drugs

There are several ethical- and human rights-based principles and dilemmas that must be considered when prioritizing fair and affordable access to expensive drugs for orphan diseases such as CF (Kacetl et al., 2020). The bioethical principles most relevant to this discussion are those of justice, beneficence, and non-maleficence (Beauchamp and Childress, 2001).

### Justice

Procedural justice speaks to the requirement for transparent and participative policymaking and resource allocation decisions and processes; distributive justice relates to the fair allocation of available resources; and social justice requires that every individual is treated with respect and dignity (Gericke et al., 2005). In the context of healthcare provision, justice refers to fair, equitable, and appropriate treatment in the light of what is due to individuals (Beauchamp and Childress, 2001; Gericke et al., 2005); most commonly interpreted as the need to provide basic healthcare for everyone (Kinney, 2014). The different models used to apply this principle, however, may be conflictual. It could be argued, for example, that CFTRm drugs constitute basic healthcare for pwCF.

The utilitarian approach to distributive justice maximizes public utility, often expressed as doing “the greatest good for the greatest number.” This approach usually forms the ethical basis of economic evaluation for healthcare expenditure. In this context, spending substantial financial resources for a rare condition such as CF could be considered unethical because it would only benefit relatively few people rather than maximizing societal benefit, and may be deemed cost-ineffective. In the face of resource constraints, funding expensive CFTRm drugs for few people may also displace cheaper healthcare interventions for more people with common conditions (Hughes et al., 2005; Ollendorf et al., 2018). In LMIC, where there is a substantial burden of disease related to communicable diseases and SDOH, it is often argued that health costs should be prioritized to meet the needs of the majority, with a focus on primary and preventive care. Treating relatively few pwCF with prohibitively expensive CFTRm is in conflict with utilitarian ethical principles of justice.

From a social justice perspective, a rights-based approach to resource management, in which all individuals within a society are entitled to a decent minimum healthcare standard, could require that treatment is made available for managing rare diseases such as CF. However, the basic right to healthcare is by its nature limited by resource constraints and is open to interpretation, even when enshrined in national legislation (Hughes et al., 2005).

It has been argued that health systems should not simply aim to maximize health gains across the entire population, but that the principle of fairness should also ensure that all people get a chance at meaningful health and wellbeing, or a “fair innings,” regardless of whether this exceeds standard measures of cost-effectiveness. Society has been shown to value this egalitarian approach to equitable distribution of resources across patients and diseases, rather than maximizing total population health (Jena and Lakdawalla, 2017; Ollendorf et al., 2018). Furthermore, society places higher value on health improvement for individuals with poorer lifetime health prospects (Dolan et al., 2005). Applying these egalitarian values upholds the moral public and policy obligation of non-abandonment of individuals with highly specialized healthcare needs, which should be considered in policy development for rationing and resource allocation for pwCF, even in LMIC settings (Landman and Henley, 1999; Hughes et al., 2005).

### Beneficence

In terms of beneficence, there is compelling real-world evidence of substantial benefits of CFTRm therapy in pwCF and responsive CFTR mutations, with reported improvements in lung function and nutrition, reduced pulmonary exacerbation rates as well as the potential to alter the trajectory of CF disease, and low mortality rates in the long term (Benden and Schwarz, 2021; Balfour-Lynn and King, 2022; Gifford et al., 2022; Keogh et al., 2022; Regard et al., 2022; Sawicki et al., 2015; Heijerman et al., 2019; Volkova et al., 2020; Carnovale et al., 2022). There is therefore clearly a conflict between the utilitarian approach to distributive justice and the principle of beneficence based on social or moral obligation (Gericke et al., 2005).

### Non-maleficence

Contrary to the ethical principle of non-maleficence, lack of access to CFTRm drugs for pwCF in LMIC may cause individual and collective harms, with a widening inequality between people with and without access to CFTRm. Delayed access to CFTRm for pwCF in LMIC has likely already had a substantial impact in this widening disparity. In 2021, Stanojevic et al. (2021) estimated that introduction of ETI in Canada in the same year would reduce the number of pwCF with severe lung disease by 60% and deaths by 15% by 2030. However, the projected benefits would be halved if access were delayed to 2025 (Stanojevic et al., 2021). For LMIC, where CF-related outcomes and mortality are likely to be substantially worse, this outlook is particularly dire (Bell et al., 2020).

People without CF may, however, also incur indirect and “invisible” harms if expensive drugs are made available to pwCF at the expense of healthcare funding for more common diseases (Ollendorf et al., 2018). Reducing the financial cost of CFTRm drugs is therefore an ethical imperative, in order to reap the benefits for pwCF whilst limiting the impact on health services. The high cost of CFTRm has been questioned recently in a study reporting that actual production costs were 90% lower than the current market prices (Guo et al., 2022b). This over-inflation of market prices for a life-saving essential drug appears unethical using the principle of distributive justice and infringes the basic human right to equal access to healthcare access, thereby widening the existing inequalities in CF care globally and leaving many pwCF behind (Guo et al., 2022b).

## Solutions to overcoming the barriers to accessing affordable CFTRm drugs

In LMIC, key barriers to achieving quality CF care are limited CF diagnosis and prevalence data and high costs of CF therapies, including CFTRm. Improving CF awareness, providing access to diagnostic tests, and creating national registries are needed to determine the actual disease burden of CF in these regions. As CFTR mutation analysis is expensive, research and development of targeted mutation panels would help identify regional- or population-specific mutations. Knowledge of regional CF epidemiology and specific mutation data would be valuable to ascertain the actual CFTRm eligibility of individual CF populations. Pharmaceutical companies should realize the potential value of investment in LMIC due to sheer numbers of patients who may benefit from these life-altering medications.

The major barrier to equitable and affordable global distribution and access to CFTRm is a prohibitive pricing strategy driven by pharmaceutical patenting practices that are protected by the World Trade Organization (WTO) agreement on Trade-Related Aspects of Intellectual Property Rights (TRIPS), established in 1995. Most countries are signatories to TRIPS, and under this agreement, countries are required to offer patents to pharmaceutical companies which typically are valid for 20 years, thus offering extended period market exclusivity without competition. While protecting intellectual property rights is fundamental, patent regulations and laws are open to abuse as highlighted by a recent US-based Association of Accessible Medicines report finding that the top 12 brands of innovative drugs on the US market are protected by a total of 848 patents (71 per drug), providing an average of 38 years without generic competition (AAM, 2022). Pharmaceutical companies point toward recuperation of research and development costs to justify extending patents beyond reasonable periods. However, the balance between rewarding innovation through global market monopoly and providing access to innovative life-altering drugs such as CFTRm is difficult to achieve due to competing interests of profit *versus* justice. The annual estimated minimum production cost of ETI by the originator company is $5,676 for one person, which is over 90% lower than the US listed price (Guo et al., 2022b). Such prohibitive pricing strategies are beyond the reach of governments and health systems in LMIC and will thus leave behind pwCF in LMIC despite CFTRm eligibility. One successful strategy to achieve that balance between protecting pharmaceutical intellectual property and providing affordable access to CFTRm in LMIC is the issuing of voluntary licenses (VL) to generic drug manufacturing companies.

Voluntary licenses are contractual agreements between patent-holding pharmaceutical companies and generic manufacturing companies, which set out terms under which generic versions of the drugs can be manufactured, marketed, and distributed in specified geographical regions (Médecins Sans Frontières Technical Briefing Document, 2020). While VL practices will reduce prices of medicines and improve access, the deals often lack transparency and come with a range of restrictive conditions that undermine access to medicines through limitations on where products can be sold and by controlling the supply of active pharmaceutical ingredients (APIs) to the licensees. Additionally, VL restrictions may include varying terms through multiple licensees and overly broad scopes of the patent that include, for example, appeals to rejected applications and geographical limitations that often exclude middle-income territories (Médecins Sans Frontières Technical Briefing Document, 2020). Excluding middle-income countries is counterproductive as they are home to 75% of the world’s population and 62% of the world’s poor and face a double burden of communicable and non-communicable diseases (Médecins Sans Frontières Technical Briefing Document, 2020). Nonetheless, VL have been successfully negotiated for human immunodeficiency virus (HIV), tuberculosis (TB), and hepatitis C drugs to the great benefit of millions of people in LMIC suffering with these conditions. More recently, Merck and Pfizer have signed VL deals with the Medicines Patent Pool (MPP) for their oral COVID-19 anti-viral medications, such as molnupiravir and nirmatrelvir (Shadlen, 2022). The MPP, established in 2010, is a United Nations-backed public health organization working to increase access to and facilitate the development of life-saving medicines in LMIC, by helping broker VL deals between originator and generic pharmaceutical companies. The major advantage of the MPP approach is that VL deals are transparent and offer pooled licensing agreements across multiple companies and territories (Shadlen, 2022). Popularity with the MPP has gained traction since the COVID-19 pandemic. We propose that this is an appropriate and attractive pathway to facilitating rapid expansion of CFTRm in LMIC. Unlike communicable diseases, such COVID-19, HIV, TB, and hepatitis C, which affect millions of people worldwide, CF is a rare orphan disease that is not prioritized in LMIC. However, CFTRm may set the precedent as advocating for VL to access innovative medicines in LMIC for a rare disease, with potential indirect benefits for other rare diseases facing similar prohibitive patent-protected pricing strategies.

Governments and civil society may revert to local legislative options to force the issuing of compulsory licenses (CLs) to licensees if originator companies are found to abuse patent conditions or impose unreasonable restrictions on patents. A CL is a license for alternative production or importation of a generic version of a patented medicine that is granted by a government or court and does require the consent of the patent holder (Médecins Sans Frontières Technical Briefing Document, 2020). Under Article 31 of the TRIPS agreement, countries are free to determine their own grounds for issuing a CL, for example, to combat anti-competitive practices and unaffordable pricing; failure to adhere to obligations with holding patents; and when public health is at stake (as was the case with COVID-19 medications and vaccines) (Shadlen, 2022). Argentina introduced legislative reforms, permitted under the TRIPS agreement, to regulate drug patent applications and promote local manufacture of generic bioequivalent formulations through issuing of CL for the benefit of public health. Argentina is therefore the only country that currently manufactures a generic ETI formulation. However, distribution and marketing to other countries where patent agreements are in place with the originator company is not permitted, and thus global access to this cheaper generic ETI is restricted (Guo et al., 2022b). Whilst CL is more likely to be granted in circumstances of public health urgencies in LMIC, such as with COVID-19, TB, or HIV, it is reasonable to argue that similar conditions should apply for a rare condition like CF, where new CFTRm is life-saving. The issuing of CL is often a protracted and costly legal process if brought forward by civil society or interest groups. Furthermore, governments that are signatories to TRIPS are reluctant to grant CL as this may lead to retaliatory trade actions and sanctions as in the case with the ongoing patent dispute between Argentina and the United States (Elliott, 2002). Reaching a deal for issuing of VL for CFTRm in LMIC is the preferred route to avoid pwCF and families being caught up in legal challenges, which may further delay accessing treatment.

Patient-led advocacy and activism is an effective and important driver for access to CFTRm drugs. The CF Buyers Club was established in the UK to procure generic CFTRm drugs from Argentina when negotiations between the UK’s National Health Service and Vertex Pharmaceuticals failed to reach an agreement over the price of Orkambi^®^ (Cohen, 2019). Similarly, the current global “Vertex Save Us” campaign is raising awareness and generating global activism for equitable access to CFTRm drugs in LMIC. These patient-led campaigns are playing a pivotal role in supporting legal steps to challenge the status of patents of CFTRm drugs in countries such as South Africa (Robbins, 2023; VSU, 2023).

## Conclusion

There are no easy solutions to correcting the global inequality and disparity in CF care, including access to CFTRm drugs. The global CF community can make a difference by raising awareness, offering clinical and diagnostic expertise, and advocating for patient rights at regional, national, and global platforms. There is strength in numbers and data, and the global CF community should unite and contribute by sharing resources and perseverant advocacy, to help initiate and direct changes in health equity, especially for pwCF who are underserved or underrepresented across the world. The prohibitive cost of CFTRm no longer affects only LMIC, but also HIC like the US, where withdrawal of the manufacturer’s funding for co-payment accumulator programs has led to unaffordable increases in out-of-pocket costs to patients and families, especially minorities. Ongoing advocacy and calls to address the unacceptably high cost of CFTRm is essential to ensure that all eligible pwCF across the world can access these life-altering drugs without discrimination (Zampoli et al., 2022; McGarry et al., 2023; Robbins, 2023).
